# Comparison of perioperative hidden blood loss for intertrochanteric fractures in the elderly by different intramedullary fixations: A randomized controlled study protocol

**DOI:** 10.1097/MD.0000000000021666

**Published:** 2020-11-25

**Authors:** Huayong Zheng, Yang Zhang, Hao Wang, Tiansheng Sun, Qicai Sun

**Affiliations:** a Department of Orthopedics, Department of orthopedics, The 7th Medical Center of PLA, Beijing; b Department of Orthopedics, Sandun Branch, Zhejiang Hospital, Zhejiang, China.

**Keywords:** complications, hidden blood loss, intertrochanteric fractures, protocol

## Abstract

**Background:**

Till date only a few studies have reported the clinical outcomes of intraoperative hidden blood loss of intertrochanteric fracture in the old people treated with various intramedullary immobilizations. The aim of the trial is to investigate the best choice for treating intertrochanteric fractures, as well as the hidden blood loss among different intramedullary fixations.

**Methods:**

This randomized, single-blind, superiority clinical trial was admitted by the Ethics Committee in our hospital (The 7th Medical Center of PLA, 20200602DM). The eligibility criteria were:

(1) Over 60 years or older;
(2) Fresh closed intertrochanteric fractures (AO 31-A2);
(3) A low- intensity trauma;
(4) With complete records of serial full blood count, including haematocrit value on admission and 72 hour after surgery.

Patients who met any of the following conditions would not be able to participate in the test: composite femoral fracture, under 65 years of ages, experience of femoral fractures, surgical contraindications, nonambulatory before the presenting injury, or presence of any other traumatic fractures. 120 participants with unstable intertrochanteric fractures, treated by Gammar nail, (n = 40), Proximal Femoral Nail Antirotation (n = 40) and Intertrochanteric Antegrade Nail (n = 40) instruments were enrolled in this research. The main outcome measures were total blood loss and hidden blood loss, which were evaluated based on the haematocrit change after the operation. The experimental data was analyzed and sorted out with SPSS program (ver.19; SPSS Inc., Chicago, IL).

**Results:**

This experiment had strict inclusive criteria and exclusive criteria and a well- regulated intervention.

**Conclusions:**

The results of this trial will provide more evidence on which technique can better treat unstable intertrochanteric fracture.

**Trial registration:**

This study protocol was registered in Research Registry (researchregistry5788).

## 1 Introduction

There are about 3 hundred thousand hip fractures in the United States every year, of which 40% to 45% occurs in the trochanteric region.^[1]^ As the population ages, the quantity of hip fractures tends to increase with reported fatality rates ranging from 15% to 30%.^[2]^ In 2040, an estimated 600,000 hip fractures will occur in the U.S. population alone, at a cost of USD 16 billion.^[3]^ In order to decrease the morbidity and treatment cost of these fractures, surgical procedures need to be optimized. However, perioperative substantial blood loss is associated with intertrochanteric fracture which cause unsatisfied outcome and systemic diseases especially for elderly individuals. These patients usually receive red blood cell transfusions to treat anemia caused by excessive hemorrhage due to the fracture or surgery. Yet, it may be associated with several side effects such as anaphylactic reaction, hemolytic reaction, infectious disease and metabolic disorders.^[4,5]^

Optimal internal fixation for the treatment of intertrochanteric fracture remains controversial. The dynamic hip screw is a kind of extramedullary fixed device which is widely used in surgical procedure.^[6,7]^ However, it shows poor clinical outcomes for treating unstable fractures, which limits its clinical use. Several intramedullary fixed devices have been used to overcome the difficulties encountered in the treatment of unstable intertrochanteric fracture. Gamma Nail and Proximal Femoral Nail Antirotation (PFNA) and Intertrochanteric Antegrade Nail (InterTan) are commonly applied with great clinical effect.^[8–10]^ For intertrochanteric fractures, the stark deficit between a large drop of Hb and seemingly small amount of perioperative visible blood loss is common among those undergoing surgery of intramedullary fixation.^[11]^ The concept of hidden blood loss (HBL) was proposed by Sehat et al. in an experiment assessing the total blood loss after total knee arthroplasty.^[12]^ HBL is a pivotal issue in aged patients with intertrochanteric fractures treated with PFNA. However, the PHBL is commonly overlooked in the patients with intramedullary fixation, owing to the relatively simple surgical procedure, short operative time and less visible blood loss. Currently, few articles have compared the HBL in aged patients with intertrochanteric fractures experiencing different intramedullary fixations. The aim of the trial is to investigate the best choice for treating intertrochanteric fractures, as well as the hidden blood loss among different intramedullary fixations.

## 2 Materials and methods

### 2.1 Study population and design

A prospective follow research design was accomplished by using data obtained from patient reports and radiological and functional recovery from regular countercheck. We examined all patients aged 65 and older between 2017 and 2018 for intertrochanteric fractures which classified Evans system (AO 31-A2). The inclusion criteria were:

(1) Fresh closed intertrochanteric fractures;
(2) A low-energy intensity;
(3) With complete records of serial full blood count, including haematocrit (Hct) value on admission and 72 hour after surgery.

Patients who met any of the following conditions would not be able to participate in the test: composite femoral fracture, under 65 years of ages, experience of femoral fractures, surgical contraindications, nonambulatory before the presenting injury, or presence of any other traumatic fractures. Data acquired from patients treated surgically with Gammar nail, PFNA or InterTan) were analyzed. Our Ethics Committee approved this prospective research (The 7th Medical Center of PLA, 20200602DM).

### 2.2 Randomization and blinding

We used a computer to randomly generate a list of three numbers of different block sizes and randomly assigned volunteers to 1 of the experimental groups in a ratio of 1:1:1. A researcher who did not take part in the trial used the website Randomization.com to generate a random distribution sequence, which was hidden in sealed opaque sequence numbered envelopes that were allocated to investigators. The surgeons, investigator, anesthetist, and nurses were all kept blinded to allocation results. 120 patients with unstable intertrochanteric fractures, treated by Gammar nail, (40), PFNA (40), or InterTan (40) instruments were recored in this experiment (Table 1). All procedures were accomplished by orthopedic surgeons with similar qualifications and years of experience in the identical institution and then was registered in research registry (researchregistry5788).

**Table 1:** Baseline characteristics of the study population.

Demographics	Gammar nail (n = 40)	PFNA (n = 40)	InterTan (n = 40)	*P* value
Age (yr)				
Male sex (no. [%] of patients)				
Weight (kg)				
Height (cm)				
Body mass index (kg/m^2^)				

### 2.3 Data collection

Using hospital records, it was possible to extract the necessary data for the present study. The following indicators were recorded from the patients’ electronic medical record: gender; age; stature; weight; kind of fracture; operation time; visual blood loss of perioperation; analytical data like hemoglobin (HB), perioperative Hct. As a consequence of discrepancies in the hospital's database, some data concerning urea and creatinin had to be converted to g/L. The Functional Recovery Score questionnaire projected by Zuckerman et al was applied to estimate functional recovery on the final follow-up examination. This procedure was done by a physiotherapist blinded to treatment allocation, who calculated the initial score on patient admission and administered the instrument again, by telephone. The Zuckerman questionnaire consists of 11 items with scores ranging from 0 to 44; a high score denotes better functional capacity. And the time to union was assessed. Radiographic healing of fractures could be regarded as the recanalization of the trabeculae or bridging callus visible on both radiograph views; delayed healing was defined as no sign of fracture healing after half a year; and nonunion was defined as the absence of bone healing after 9 months after surgery.

### 2.4 Blood loss calculation

The amount of blood loss was evaluated according to the change in Hct level and the expected total blood volume. Total blood volume (TBV) was projected by the Nadler's formula, considering the gender of the patient, as shown below: Women: BV (l) = height (m)^3^ × 0.3561 + weight (kg) × 0.03308 + 0.1833. Men: BV (l) = height (m)^3^ × 03669 + weight (kg) × 0.03219 + 0.6041. Total red blood cell loss = TBVpreop × (Hctpreop – Hctpostop). Total blood loss (l) = Red blood cell loss/Hctpreop. The hidden blood loss = Total blood loss–visual blood loss.^[13]^

Perioperative blood loss was measured by the previous formula and Gross's formula.^[14]^ The visual blood loss included the quantity of liquid in the suction bottle minus the amount of liquid for flushing the wound, and the net weight of the gauze, gauze pad, and the surgical towel. Laboratory tests were carried out preoperatively and 1 days postoperatively in the same laboratory. The administration standard of blood transfusion was hemoglobin value < 70 g/L, or hemoglobin value was < 80 g/L with anemia symptoms occurred. Management of fluid electrolyte balance and blood transfusion was determined by the anesthesia team, who were blinded to the kind of fixator.

### 2.5 Statistical analysis

Descriptive statistics were applied to display the basic features of the patient groups. One-way ANOVA (Kruskal-Walli Test) was used to analyze the difference of blood loss among the 3 surgical methods. Mann-Whitney *U* test was applied to compare the 2 methods. When *P* value is less than .05, it means that the test has statistical significance. The experimental data was analyzed and sorted out with SPSS software (ver.19; SPSS Inc., Chicago, IL).

## 3 Results

The results will be shown in Table 1 and Table 2.

**Table 2:** Comparison of perioperative clinical outcome between the groups.

Outcomes	Gammar nail (n = 40)	PFNA (n = 40)	InterTan (n = 40)	*P* value
Surgical blood loss (mL)				
Total blood loss (mL)				
Transfusion rate (%)				
Visible blood loss (mL)				
Hidden blood loss (mL)				
Haematocrit values (%)				
Admission (preoperative); Final (72 h post-operative)				
Haemoglobin values (g/L); Admission (preoperative); Final (72 h post-operative)				

## 4 Discussion

The treatment of unstable intertrochanteric fractures in the aged patients and the postoperative recovery has been a challenge for the orthopaedic surgeons.^[15,16]^ Compared with patients with femoral neck fracture, patients with intertrochanteric fracture have more blood loss and higher blood transfusion rate. Perioperative blood loss was associated with increased fatality rate, infection, deep venous thrombosis, renal and cardiac decompensation and dysfunction. Therefore, these conditions must be considered in the treatment of intertrochanteric fractures.^[17]^ Reducing the total blood loss was a major problem in the treatment of intertrochanteric fractures, and how to prevent varus displacement, screw cutting and bone healing was a further problem to be solved.^[18]^ The HBL frequently occurs after intramedullary nailing surgery. This may be caused by numerous factors.

(1) partial injury related to HBL occurs before admission in fact;
(2) the anti-coagulant treatment after surgery may further increase the HBL;
(3) insufficient hemostasis may cause continued postoperative blood.^[19,20]^

There are still some limitations in the research. First of all, short follow-up time and small sample size leaded to weak statistical ability of final data. Second, the main results are based on the calculation of a few clinical measurement results, and there are some measurement errors. During hospitalization and operation, rehydration can affect hemoglobin measurement. Moreover, the kind and volume of infusion for each patient were not recorded normatively. High quality of RCTs with large sample size is required to convince our results.

## 5 Conclusion

The results of this trial will provide more evidence on which technique can better treat unstable intertrochanteric fracture.

## Author contributions

Qicai Sun and Tiansheng Sun planned the study design. Hao Wang reviewed the study protocol. Yang Zhang will recruit participants and collect data. Huayong Zheng wrote the manuscript. All of the authors have read, commented on, and contributed to the submitted manuscript.

**Data curation:** Yang Zhang.

**Formal analysis:** Hao Wang.

**Resources:** Tiansheng Sun.

**Writing – original draft:** Huayong Zheng.
